# Olanzapine treatment effectively relieves breakthrough chemotherapy-induced nausea and vomiting: a real-world experience

**DOI:** 10.1186/s40780-023-00293-y

**Published:** 2023-08-01

**Authors:** Akihiro Uchiike, Haruka Kono, Katsuhiro Miura, Tatsuya Hayama, Daisuke Tsutsumi, Shinya Tsuboi, Susumu Ohtsuka, Shinji Hidaka

**Affiliations:** 1 Nihon University Itabashi Hospital Tumor Center, 30-1 Oyaguchikamicho, Itabashi, 173-8610 Tokyo Japan; 2 Department of Pharmacy, Nihon University Itabashi Hospital, Tokyo, Japan; 3 Department of Pharmaceutical Regulatory Science, School of Pharmacy, Nihon University, Chiba, Japan; 4 Division of Hematology and Rheumatology, Department of Medicine, Nihon University School of Medicine, Tokyo, Japan; 5 Present Address: Department of Medical Oncology, Toranomon Hospital, Tokyo, Japan

**Keywords:** Chemotherapy-induced nausea, Highly emetogenic chemotherapy, Olanzapine

## Abstract

**Background:**

Olanzapine treatment prevents chemotherapy-induced nausea and vomiting (CINV) in patients receiving highly emetogenic chemotherapy (HEC). However, its role in the secondary prevention of breakthrough CINV in real-world cancer care should be further evaluated.

**Method:**

We conducted a retrospective study on patients receiving olanzapine to prevent breakthrough CINV refractory to standard antiemetic therapy. The major outcome was improvement in CINV, defined as any downgrade in CINV symptoms, according to the Common Terminology Criteria for Adverse Events. Comprete response was defined as no symptoms in CINV, i.e., Grade 0. These outcomes were compared in the HEC versus non-HEC groups and the standard- (5 or 10 mg/day) versus low- (2.5 mg/day) dose groups. The other outcome measurement was adverse events (AEs).

**Results:**

We analyzed 127 patients, including 92 women, with a median age of 50 years (range: 19–89 years). Baseline CINV severity was grade 1, 2, and 3 in 18%, 69%, and 13% of the patients, respectively. After prophylaxis with olanzapine at doses of 2.5, 5, or 10 mg/day, improvement was observed in 105 (83%) patients, with a complete response in 42 patients (33%). The improvement and complete remission rates for the HEC (n = 96) and non-HEC (n = 31) groups were 86% and 71% (p = 0.048) versus 38% and 19% (p = 0.062), respectively. The rates for the standard- (n = 98) and low- (n = 29) dose groups were 86% and 82% (p = 0.568) versus 28% and 52% (p = 0.015), respectively. Thirty-four patients (27%) experienced olanzapine-related AEs, mainly somnolence (n = 28). Grade 3 or higher AEs were not observed.

**Conclusion:**

Our study results support the clinical application of olanzapine for the secondary prevention of breakthrough CINV.

## Background

Chemotherapy-induced nausea and vomiting (CINV) is a prominent adverse event (AE) that deteriorates the quality of life (QOL) of patients receiving anticancer chemotherapy. In addition, CINV can induce postponement of, reduction in, or discontinuation of effective chemotherapy treatment [1]. Despite modern oncology practice involving the use of prophylactic antiemetic therapies per authorized guidelines, up to half of the patients still experience CINV [2][3]. Therefore, improving the control of CINV is critical for improving patient QOL and ensuring successful anticancer treatment [4].

Olanzapine, a second-generation antipsychotic that blocks serotonin type-2 5-hydroxytryptamine receptors (5-HT) and dopamine D2 receptors, has demonstrated efficacy in preventing CINV, especially in patients receiving highly emetogenic chemotherapy (HEC) [5] [6]. In a pivotal randomized controlled study of 380 patients undergoing HEC, the addition of olanzapine to the standard three-drug regimen, consisting of palonosetron, aprepitant, and dexamethasone, was found to significantly reduce chemotherapy-induced nausea compared with placebo. This was demonstrated in the first 24 h after chemotherapy (74% vs. 45%, p = 0.002), 25–120 h after chemotherapy (42% vs. 25%, p = 0.002), and overall 0–120-h period (37% vs. 22%, p = 0.002) [7].

As olanzapine is widely used in cancer treatment for the prevention of CINV, its clinical utility has been widely debated. Contrary to the essential role of olanzapine in the primary prevention of CINV, few studies have evaluated its efficacy in the secondary prevention of breakthrough CINV with limited numbers of patients [8][9][10]. Similarly, evidence of the effect of olanzapine on moderate emetogenic chemotherapy (MEC) or low emetogenic chemotherapy (LEC) remains insufficient. Furthermore, the dose of olanzapine has aroused the interest of oncologists because its AEs, such as drowsiness and lightheadedness, are problematic for both patients and their clinicians. To improve our understanding of the potential roles of olanzapine in real-world oncology practice, we conducted an observational study to evaluate the efficacy and safety of olanzapine at various doses in patients who experienced breakthrough CINV, regardless of the risk of chemotherapy regimens.

## Methods

### Study design

This was an exploratory, retrospective, observational study conducted at the Nihon University Itabashi Hospital Tumor Center, located in Tokyo, Japan. The inclusion criterion was patients who orally received olanzapine for the secondary prevention of breakthrough CINV between January 2014 and March 2020 at Nihon University Itabashi Hospital. Secondary prevention was defined as the use of regular oral olanzapine for antiemetic purposes, beginning with a subsequent course of cancer chemotherapy with standard antiemetic therapy using corticosteroids, 5-TH3 receptor antagonists, with or without aprepitant. Patients younger than 18 years or those with pre-existing diabetes were excluded. First, study candidates were extracted from the patient database of our tumor center. Then, electronic medical records were reviewed for eligibility for the study protocol by expert oncology physicians and pharmacists.

### Treatment regimen and study drug

In principle, the patients received olanzapine on days 1–4 before bedtime, but the duration of treatment was according to the physician’s discretion and the patient’s symptoms. Similarly, dosage of olanzapine was determined on a case-by-case basis at 10 mg, 5 mg, or 2.5 mg daily. We categorized each chemotherapy regimen as HEC, MEC, or LEC according to the guidelines of the American Society of Clinical Oncology [11]. For a simplified comparison, we considered MEC or LEC as “non-HEC” regimens. Similarly, we considered 10 mg/day or 5 mg/day of olanzapine as “standard doses” and 2.5 mg/day as a “low dose.” These groupings were to avoid multiple comparisons, which may complicate statistical analysis. According to the institution’s guidelines, treating physicians managed to prevent CINV based on the risk of the chemotherapy administered to patients. Olanzapine was prescribed to patients who experienced any grade of CINV in the latest chemotherapy cycle, despite receiving standard antiemetic therapy. The indication of olanzapine was determined at the discretion of oncology physicians and pharmacists. For these patients, CINV symptoms and olanzapine-associated AEs were carefully recorded during the following chemotherapy cycle to evaluate the efficacy and toxicity of olanzapine, both in the acute (0–24 h after chemotherapy) and delayed (24–120 h after chemotherapy) phases.

### Study endpoints

Treatment efficacy was categorized as either improvement, defined as any reduction in the severity (grade) of CINV symptoms, or failure, defined as equivalent or upgrades in symptoms, or discontinuation of olanzapine for any reason. The primary endpoint was the rate of improvement in CINV in all patients. The secondary endpoints of the study included the complete remission rate for all patients, which was the proportion of patients who did not experience CINV. The study also aimed to determine the improvement and complete remission rates for each severity grade of CINV, as well as for HEC and non-HEC regimens, and for standard and low doses of the treatment drug. Additionally, the study aimed to assess any adverse effects related to the treatment drug olanzapine. CINV and olanzapine-related AEs were graded according to the Common Terminology Criteria for Adverse Events Version 4.0 CTCAE ver4.0 (https://evs.nci.nih.gov/ftp1/CTCAE/About.html#:~:text=CTCAE_4.03_2010%2D06%2D14_QuickReference_5x7.pdf).

### Statistical analysis

Continuous variables were presented as means or medians, where appropriate. Categorical variables are presented as counts and percentages. Intergroup differences were compared using the χ^2^ test for categorical variables, with a p-value < 0.05 defined as statistically significant. We did not calculate the targeted sample size because no previous studies evaluated olanzapine with a similar design. However, based on the number of patients prescribed olanzapine in our center during the study period, the estimated sample size of 100 was considered enough to detect the efficacy of olanzapine in secondary prevention. All analyses were performed using the JMP software version 14.3.0 (SAS Institute, Cary, NC, USA).

### Ethics approval

This study was conducted in accordance with the principles of the Declaration of Helsinki. All patients provided informed consent for chemotherapy and supportive therapies, including CINV prophylaxis. The Nihon University Itabashi Hospital Clinical Research Judging Committee approved this study for data collection, analysis, and publication on April 15, 2020 (RK-200414-6).

## Results

We included 168 patients but excluded 41 from the primary analysis because of different reasons for olanzapine use (n = 35) or poor records of CINV grades (n = 6). Accordingly, we analyzed data from 127 patients with a median age of 50 years. The treated cancer types were lymphoma in 24 (19%) patients, lung cancer in 19 (15%), breast cancer in 18 (14%), cervical cancer in 17 (12%), leukemia in seven (6%), head and neck cancer in seven (6%), sarcoma in seven (6%), endometrial cancer in four (3%), seminoma in four (3%), and other types of cancer in 20 (16%). Among these, 96 and 31 patients received HEC and non-HEC (MEC or LEC) regimens, respectively. Before the addition of olanzapine, the severity of CINV was grade 1 in 23 patients, grade 2 in 88, and grade 3 in 16. The prescribed dose of olanzapine was 10 mg/day in four patients, 5 mg/day in 94, and 2.5 mg/day in 29 (Table 1). The median duration of the olanzapine treatment was four days (range 1–10 days).

**Table 1:** Patient characteristics

Characteristics	Total (N = 127)
Male/female	35/92
Median age (range)	50 (19–89) years
Median body weight (range)	53 (35–87) kg
Chemotherapy risk for CINV^a^	
HEC^b^ regimens, n (%)	96 (76%)
MEC^c^ regimens, n (%)	25 (20%)
LEC^d^ regimens, n (%)	6 (5%)
Baseline CINV grades	
Grade 1, n (%)	23 (18%)
Grade 2, n (%)	88 (69%)
Grade 3, n (%)	16 (13%)
Grade 4, n (%)	None
Olanzapine doses	
10 mg/day, n (%)	4 (3%)
5 mg/day, n (%)	94 (74%)
2.5 mg/day, n (%)	29 (23%)

^a^ chemotherapy-induced nausea and vomiting

^b^ highly emetogenic chemotherapy

^c^ moderate emetogenic chemotherapy

^d^ low emetogenic chemotherapy

**Fig. 1:**
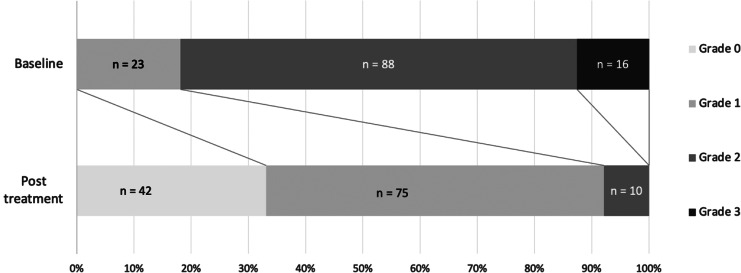
Alterations in the proportions of chemotherapy-induced nausea and vomiting grades

CINV severity during the initial chemotherapy course with olanzapine was grade 0 in 42 patients (33%), grade 1 in 75 (59%), and grade 2 in 10 (8%) (Fig. 1). The improvement rate for all patients was 83% (n = 105), with a complete remission rate of 33% (n = 42). Treatment failure, i.e., no reduction in CINV grade, was observed in 22 (17%) patients. The number of patients who reported any grades of nausea and vomiting in the acute and delayed phases after olanzapine prophylaxis was generally lower than that before prophylaxis (Table 2). The improvement and complete remission rates for HEC versus non-HEC regimens were 86% and 71% (p = 0.048) versus 38% and 19% (p = 0.062), respectively. The improvement and complete remission rates for the standard-dose group compared with those for the low-dose group were 82% and 86% (p = 0.568) versus 28% and 52% (p = 0.015), respectively (Table 3).

**Table 2:** Alterations of the number of patients with nausea and vomiting in acute and delayed phases

	Nausea (n = 127)		Vomiting (n = 127)	
	Acute (0–24 h)	Delayed (24–120 h)	Acute (0–24 h)	Delayed (24–120 h)
Baseline	33 (26%)	127 (100%)	2 (2%)	21 (17%)
Post-treatment	11 (9%)	83 (65%)	1 (1%)	6 (5%)

**Table 3:** Comparisons of improvement and complete remission rates among the patient groups

	HEC (n = 96)	non-HEC (n = 31)	p-value		Standard doses (n = 98)	low dose (n = 29)	p-value
Improvement, n (%)	83 (86%)	22 (71%)	0.048		80 (82%)	35 (86%)	0.568
Complete remission, n (%)	36 (38%)	6 (19%)	0.062		27 (28%)	15 (52%)	0.015

AEs related to olanzapine were observed in 34 (27%) patients. The most common AEs were somnolence (n = 28), dizziness (n = 3), fatigue (n = 1), tremor (n = 1), restless leg syndrome (n = 1), fatigue (n = 1), hallucination (n = 1), and tremor (n = 1). However, the severity of all AEs was either grade 1 or 2 (Table 4). The incidence of AEs between the standard- and low-dose groups was not significantly different (28% vs. 24%, p = 0.715).

**Table 4:** Olanzapine-related adverse events

Adverse events	Total (n = 127)		
	All grades	Grade 1	Grade 2
Somnolence	28	13	15
Dizziness	3	2	1
Fatigue	1	0	1
Tremor	1	1	0
RLS^a^	1	1	0
Hallucination	1	1	0

^a^restless leg syndrome.

## Discussion

This study demonstrated the remarkable efficacy of olanzapine as a rescue medication for patients who experience nausea and vomiting despite receiving the standard primary prevention against CINV. The overall and complete remission rates of CINV of 83% and 33%, respectively, consolidate the excellent efficacy of olanzapine as a secondary prevention drug for CINV, which had not been extensively evaluated until recently. Navari et al. performed one of the few published randomized, double-blind studies in this setting. They demonstrated the superiority of olanzapine (10 mg once daily for 3 days orally) over metoclopramide (10 mg three times daily for 3 days orally), either added to the conventional three-drug regimen, for patients with breakthrough CINV after they received HEC (≥ 70 mg/m^2^ cisplatin or doxorubicin-cyclophosphamide-containing regimens). In this study, the proportion of patients without nausea during the 72-h observation period was significantly higher in the olanzapine group compared with the metoclopramide group (65% [38 of 56] and 23% [12 of 52], p < 0.01) [8]. Chanthawong et al. performed a phase 2 study that evaluated olanzapine (5 mg every 12 h for two doses orally) in patients who received HEC and experienced breakthrough emesis despite antiemetic prophylaxis with ondansetron, corticosteroids, and metoclopramide. The complete responses for breakthrough emesis and nausea among 46 evaluated patients during a 24-h observation period were 61% and 50%, respectively [9]. Vig et al. performed a retrospective analysis of 33 patients with breakthrough CINV refractory to dopamine antagonists and benzodiazepines, who received at least one dose of 5–10 mg oral olanzapine. They concluded that the overall success rate, defined as lower than five emesis events in 24 h or < 10 cumulative doses of rescue antiemetics, was 70%. Post hoc analysis showed that efficacy of olanzapine was observed regardless of sex, degree of chemotherapy emetogenicity, number of prophylactic antiemetics, or age [10]. Since these previous studies were limited by the chemotherapy regimens or olanzapine doses, our results provide new insights into the secondary prevention of breakthrough CINV using olanzapine.

Notably, we found that olanzapine was not as effective in patients receiving non-HEC as in those receiving HEC, although statistical differences were marginal. This finding was contrary to the favorable response observed in the HEC group, which was expected based on the results of a previous study [8]. In a randomized, double-blind, placebo-controlled study evaluating the use of olanzapine with palonosetron and dexamethasone in 56 patients receiving MEC, the reduction in CINV incidence was limited despite better QOL in the olanzapine group [12]. In this context, a recent systematic review of olanzapine for CINV described the paucity of data on the utility of olanzapine in MEC regimens [13]. However, these previous studies evaluated olanzapine for MEC regimens mostly in a primary prophylaxis setting. Although the prophylactic efficacies of olanzapine for breakthrough CINV in our patients in the non-HEC group were not as satisfactory as those in the HEC group, substantial improvements in CINV symptoms were found in the non-HEC group. This result prompts the further exploration of effective treatments for breakthrough CINV in these populations.

Another remarkable finding of the study was the acceptable efficacy of a low dose (2.5 mg/day) of olanzapine compared with that of the standard doses (5 or 10 mg/day). The complete response rate in the low-dose group was unexpectedly higher than that in the standard-dose group. In treating schizophrenia, the standard dose of olanzapine is 5–20 mg/day, since an adequate plasma olanzapine concentration is required to improve the psychiatric symptoms [14] [15]. Therefore, the earliest studies used 10 mg/day as the experimental dose of olanzapine to prevent CINV [16] [17]. However, the sedative side effects of olanzapine are particularly problematic for patients who do not have psychotic symptoms. After exploration of less toxic treatment regimens that can retain sufficient antiemetic effect in several randomized studies, 5 mg/day olanzapine is now considered the new standard for the prevention of CINV [18] [19]. However, in our daily oncology practice, we observed that even 5 mg/day of olanzapine treatment could produce sedative effects in patients. Whereas the approved dose of olanzapine for CINV is 5–10 mg daily in Japan, we prescribe 2.5 mg/day olanzapine in cases where the side effects of olanzapine are anticipated, such as in patients with low body weight or elderly individuals. We also hypothesized that olanzapine at lower doses could effectively occupy the D2 receptor to prevent CINV [20]. Thus, 2.5 mg/day of olanzapine can be a reasonable alternative dose for carefully selected patients with breakthrough CINV.

Given the retrospective nature of our study that was conducted at a single institution, several concerns should be addressed before interpreting the results. First, the sample size was not powered to identify intergroup differences. In addition, our study population was relatively heterogeneous in terms of cancer type, age, disease advancement, treatment risks for CINV, and baseline antiemetic therapies. Therefore, well-designed prospective studies should be performed to verify our results. Next, based on case-by-case decisions, the treatment regimen of olanzapine for breakthrough CINV was not appropriately organized according to dosage or duration. As a result, the treatment schedule for olanzapine was not uniform in our study, possibly affecting the efficacy or toxicity of the medication. Finally, the effectiveness of 2.5 mg/day of olanzapine treatment in secondary or primary prevention of CINV requires prospective studies.

## Conclusions

In conclusion, secondary prophylaxis with olanzapine for breakthrough CINV effectively rescued patients from symptoms. The benefits of olanzapine treatment were significant in patients receiving HEC regimens. The standard dose of 5 mg/day of olanzapine is the most recommended, but 2.5 mg/day of olanzapine may be a more suitable option for a subset of patients who are likely to be susceptible to the toxicities of the treatment.

## Data Availability

Anonymized data from this study are available upon reasonable request to the corresponding author.

## List of abbreviations

CINV: chemotherapy-induced nausea and vomiting
HEC: highly emetogenic chemotherapy
AE: adverse event
QOL: quality of life
MEC: moderate emetogenic chemotherapy
LEC: low emetogenic chemotherapy
